# Supplementary material for: Paclobutrazol Promotes Root Development of Difficult-to-Root Plants by Coordinating Auxin and Abscisic Acid Signaling Pathways in Phoebe bournei
Source: Int J Mol Sci. 2023 Feb 13;24(4):3753. doi: 10.3390/ijms24043753 (PMC9958905; doi:10.3390/ijms24043753)
Supplement: Supplementary file 1 [file ijms-24-03753-s001.zip › ijms-2138678-supplementary.pdf]

Paclobutrazol promotes root development of difficult-to-root plants by coordinating auxin and abscisic acid signaling pathways in *Phoebe bournei*

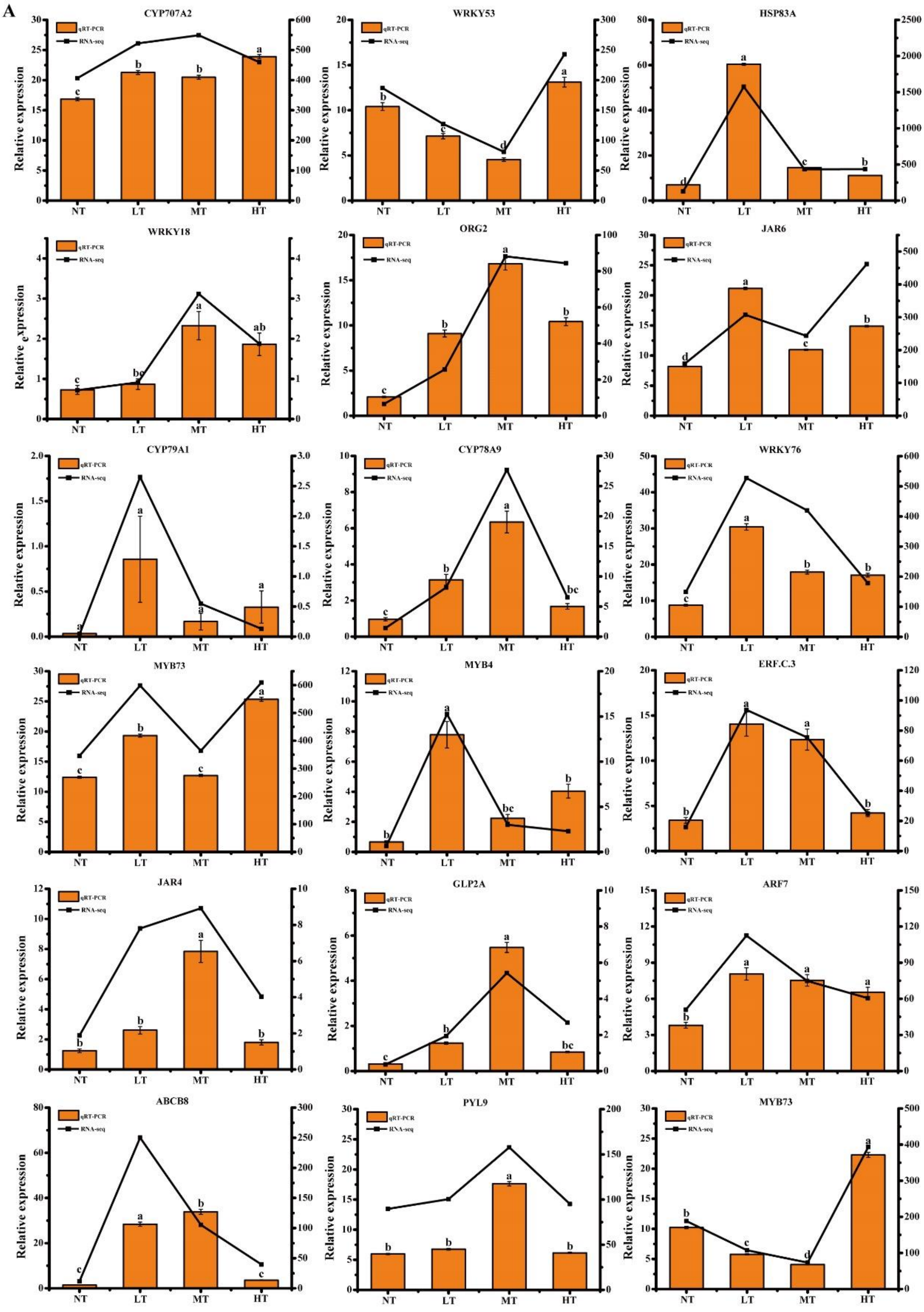

Figure S1. Comparison of the expression levels of the randomly selected genes by qRT-PCR and RNA-seq in *P. bournei* roots. The orange histograms represent qPCR results (2-ΔΔCt), and the broken line graphs represent RNA-seq results (FPKM). All data showed the average value of three biological replicates. Different lowercase letters indicate significant differences under four PBZ treatments (ANOVA, $P<0.05$).

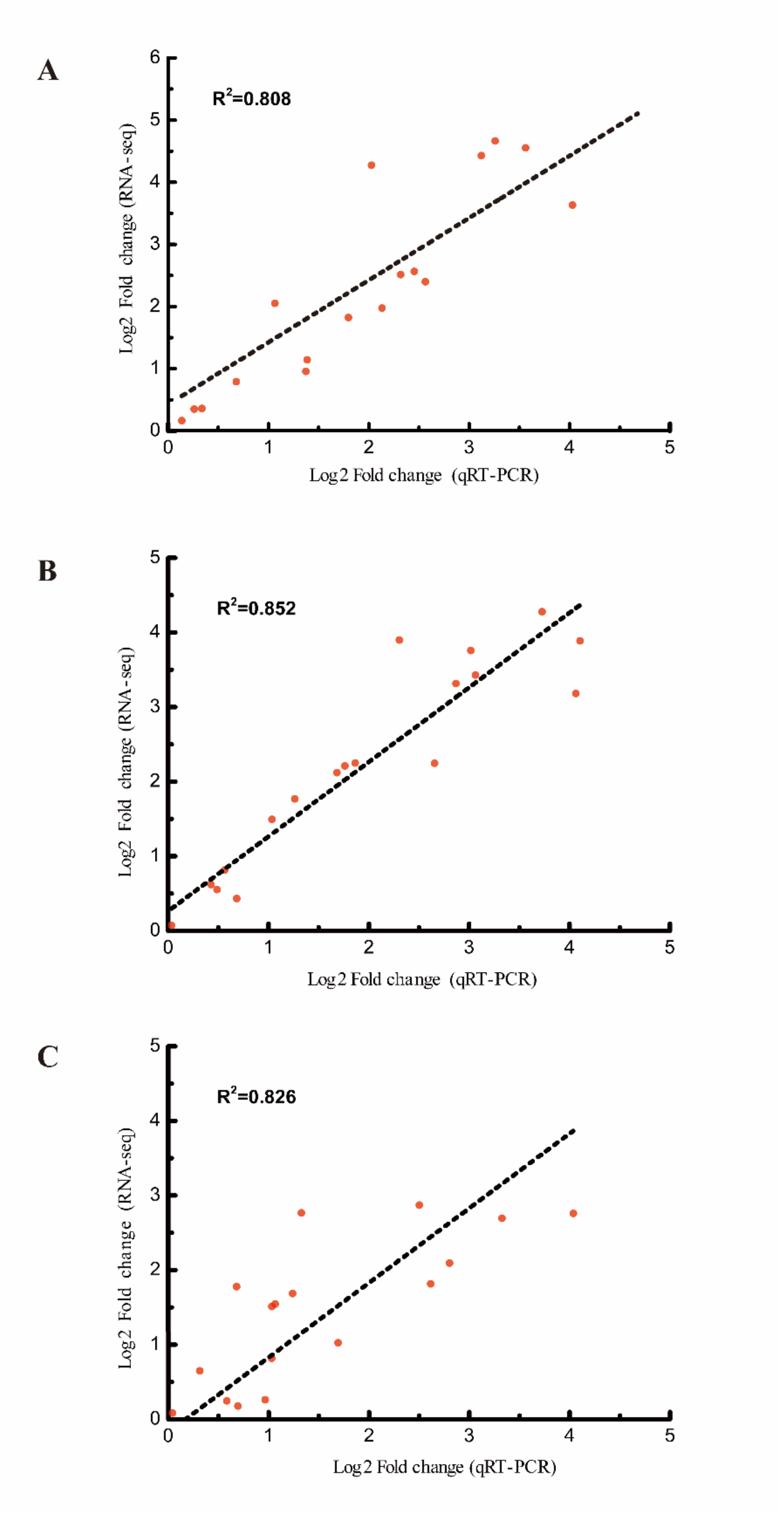

Figure S2. Correlation plot of the RNA-Seq and qRT-PCR results. (A) A linear regression analysis of the fold-change (NT-vs-LT) between the DGEs and the qRT-PCR; (B) A linear regression analysis of the fold-change (NT-vs-MT) between the DGEs and the qRT-PCR. (C) A linear regression analysis of the fold-change (NT-vs-HT) between the DGEs and the qRT-PCR. The above results were calculated using log2 fold change value. Total RNA was extracted from three replicated samples individually for each treatment.

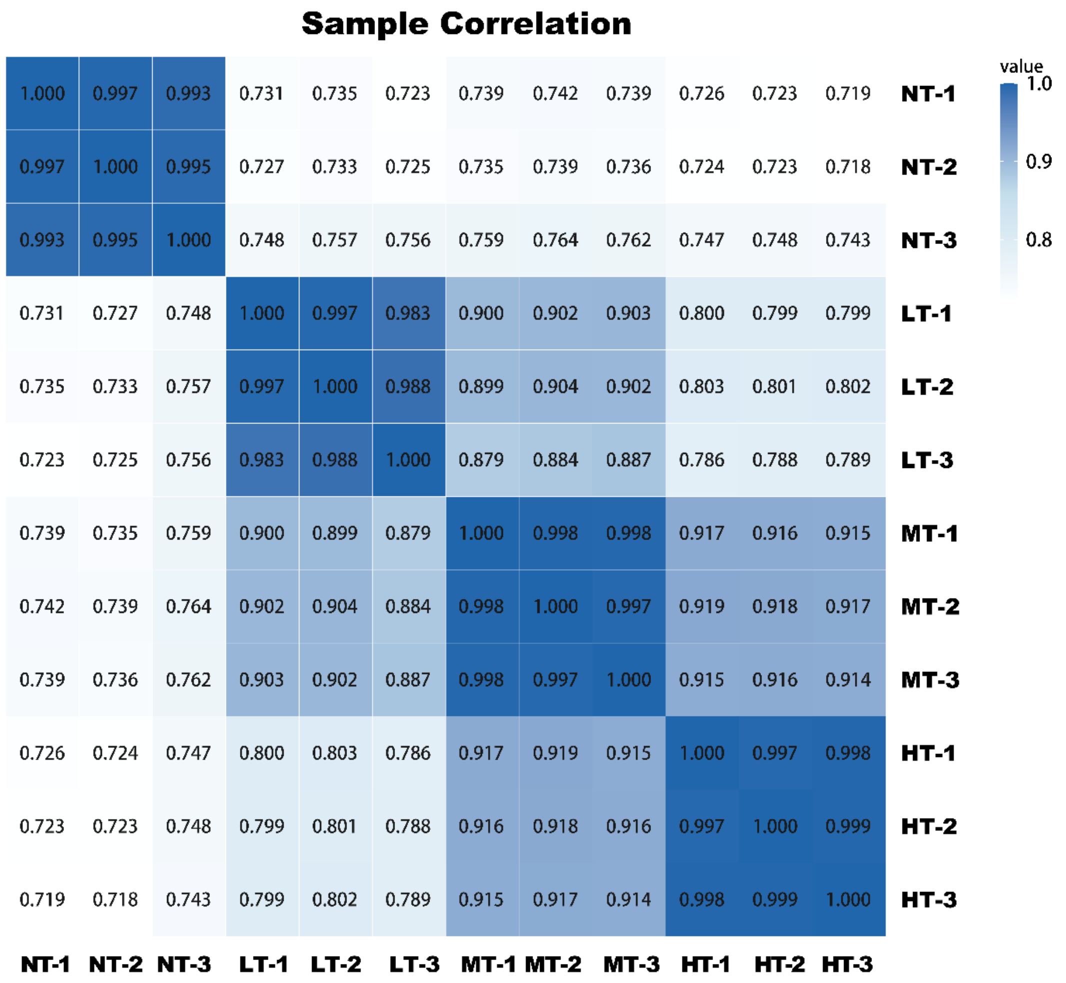

Figure S3. Analysis of correlation heatmap among each sample. Representative images of 3-month-old seedlings grown under control (NT), 0.6mg/L(LT), 6mg/L(MT), 60mg/L(HT) treatment of paclobutrazol.

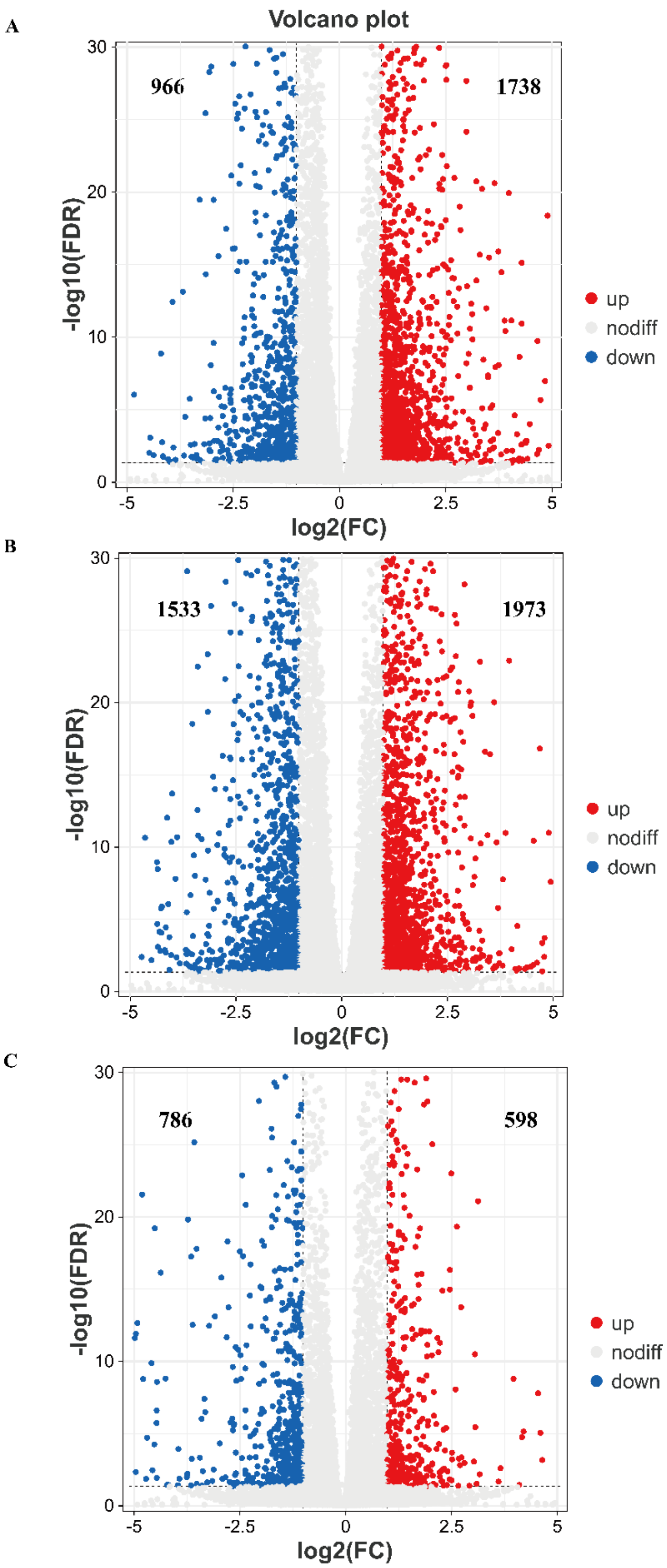

Figure S4. Volcano plot showed the numbers of up- and down-regulated DEGs in roots under different treatments. (A) LT-VS-MT, (B) LT-VS-HT, (C) MT-VS-HT.

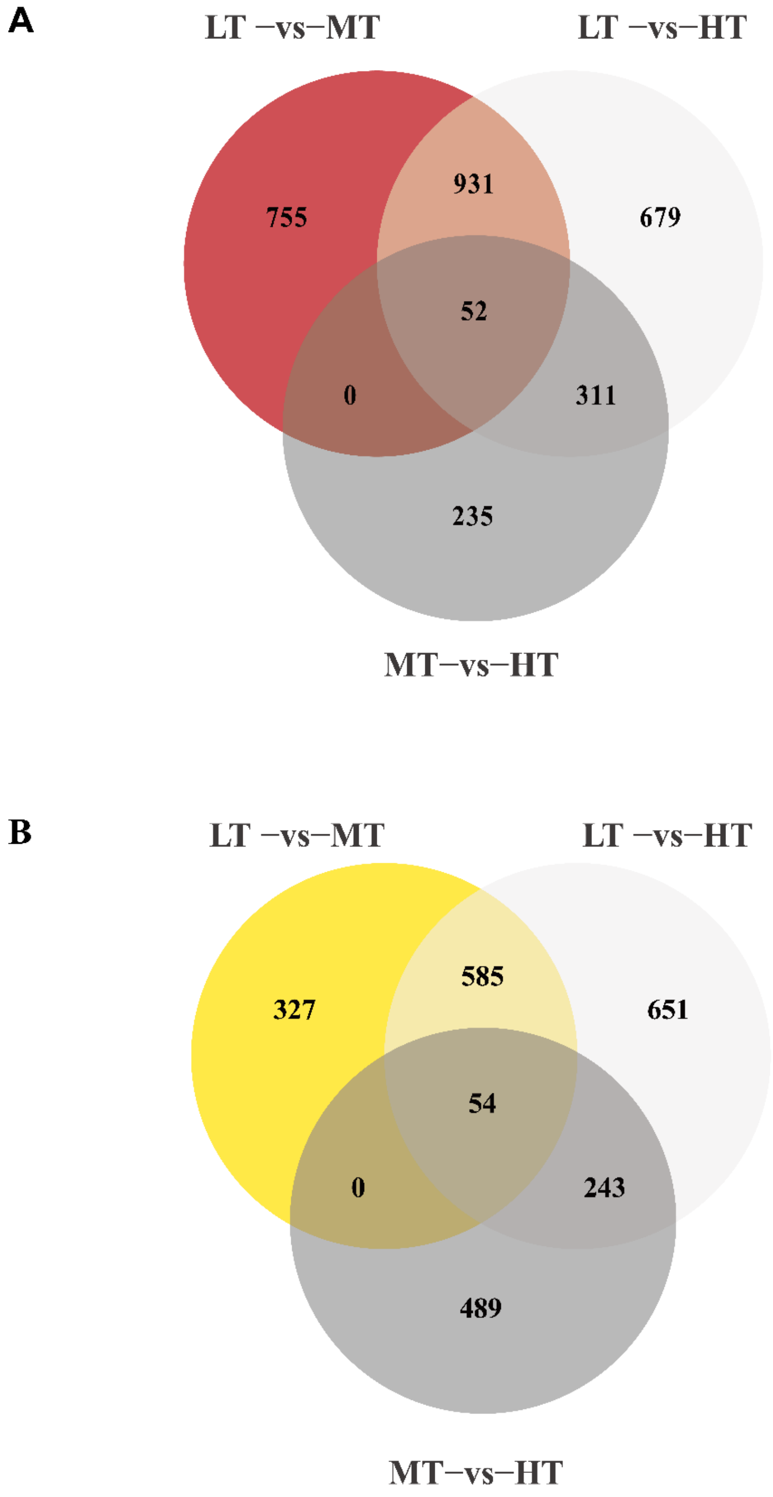

Figure S5. Venn diagrams showing the numbers of common DEGs and the overlapping sets obtained across treated groups, including (A) Up-regulated DEGs and (B) down-regulated DEGs in roots.

A

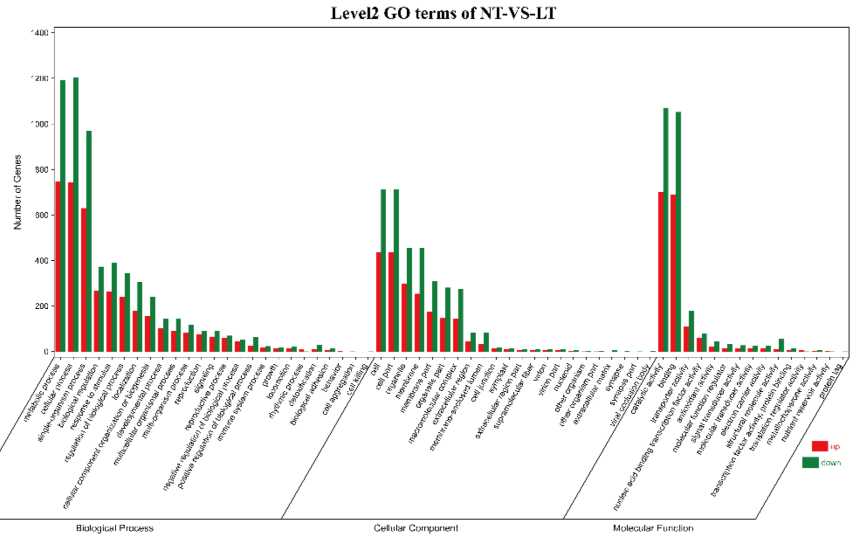

B

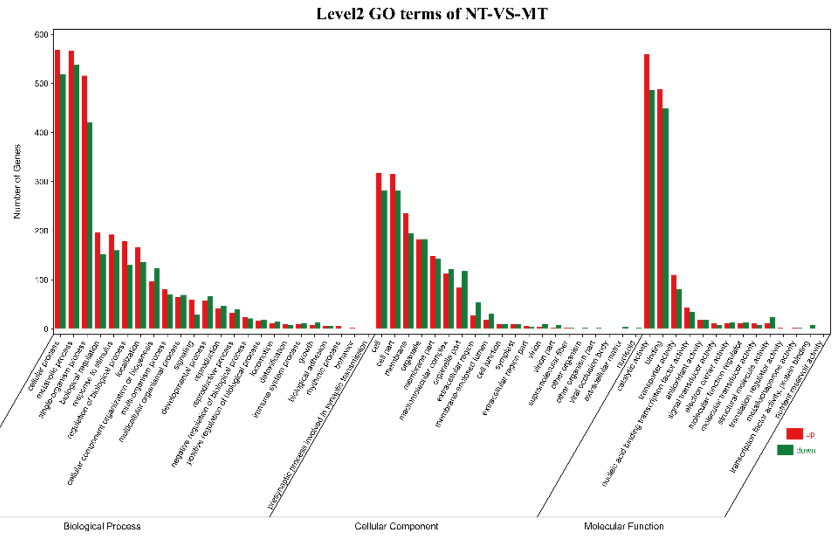

C

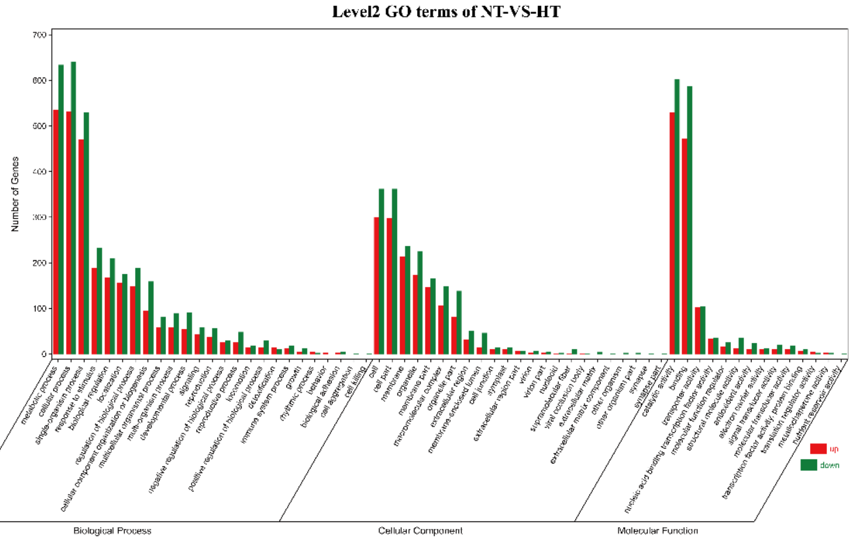

Figure S6. Histogram of GO enrichment classification in paclobutrazol treatments, including (A) NT-vs-LT, (B) NT-vs-MT and (C) NT-vs-HT.

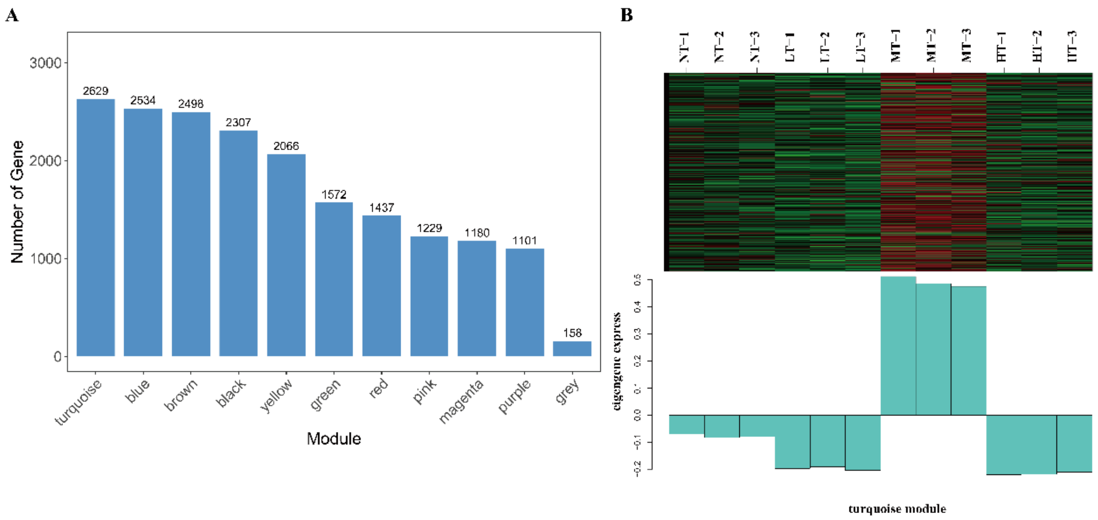

Figure S7. (A) Number of genes and (B) expression profile of modules associated with root traits with paclobutrazol treatments.

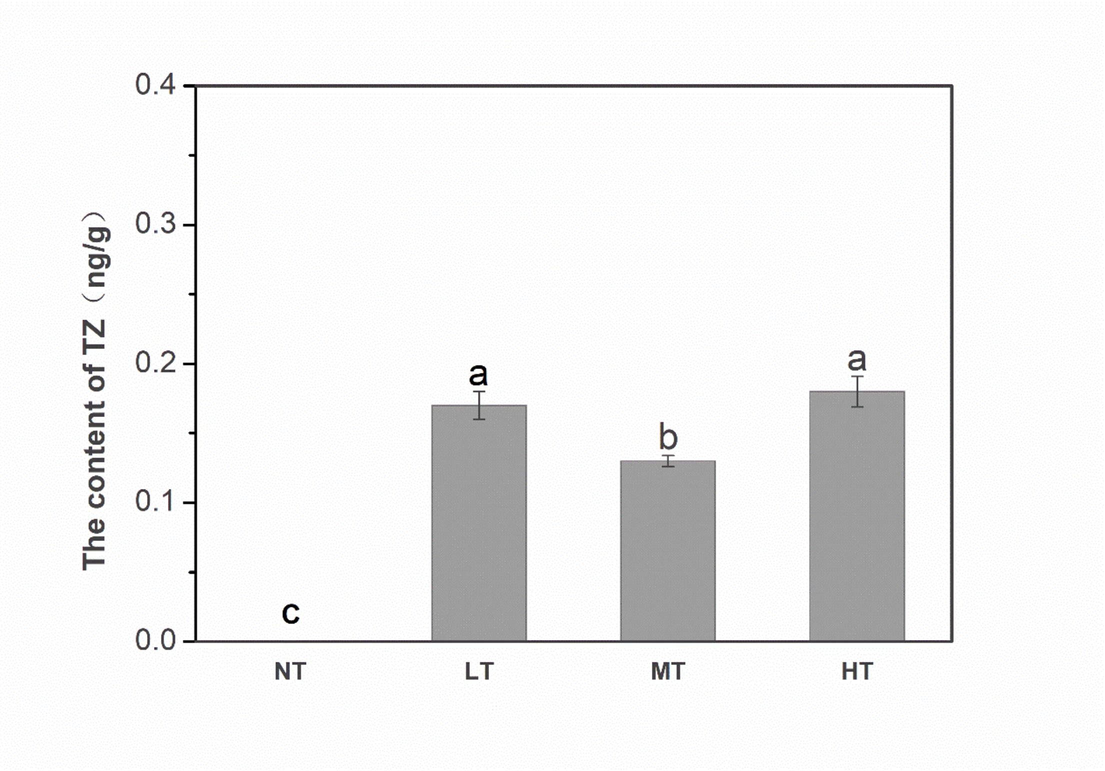

Figure S8. Effects of different concentrations of paclobutrazol on the content of TZ in *P. bournei* roots. Different lowercase letters indicate significant differences under four PBZ treatments (ANOVA, $p < 0.05$).
