# Supplementary material for: Paclobutrazol Promotes Root Development of Difficult-to-Root Plants by Coordinating Auxin and Abscisic Acid Signaling Pathways in Phoebe bournei
Source: Int J Mol Sci. 2023 Feb 13;24(4):3753. doi: 10.3390/ijms24043753 (PMC9958905; doi:10.3390/ijms24043753)
Supplement: Supplementary file 1 [file ijms-24-03753-s001.zip › Supplementary information_LC-MS profiles.pdf]

Original research

Paclobutrazol promotes root development of difficult-to-root plants by coordinating auxin and abscisic acid signaling pathways in *Phoebe bournei*

LC-MS profiles of representative samples

1. Sample raw data

Analyte: IAA 1

NT-1

RT (Exp. RT):	6.35 (6.35) min
Calculated Conc:	1.84 ng/mL
Area:	67300.
Area Ratio:	9.35e-002
Sample Type:	(Unknown)

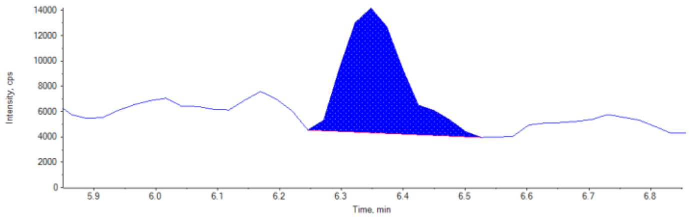

NT-2

RT (Exp. RT):	6.34 (6.35) min
Calculated Conc:	1.84 ng/mL
Area:	71200.
Area Ratio:	9.37e-002
Sample Type:	(Unknown)

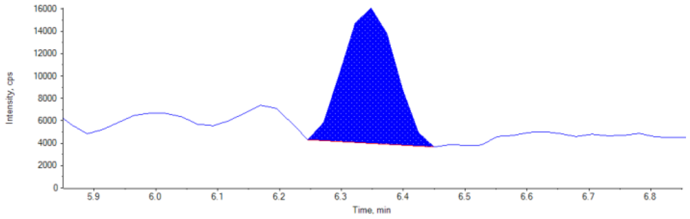

NT-3

RT (Exp. RT):	6.35 (6.35) min
Calculated Conc:	2.06 ng/mL
Area:	80700.
Area Ratio:	1.05e-001
Sample Type:	(Unknown)

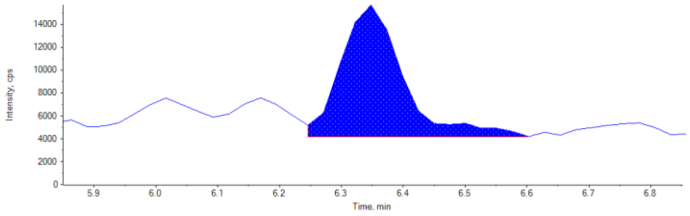

LT-1

RT (Exp. RT):	6.35 (6.35) min
Calculated Conc:	7.28 ng/mL
Area:	206000.
Area Ratio:	3.68e-001
Sample Type:	(Unknown)

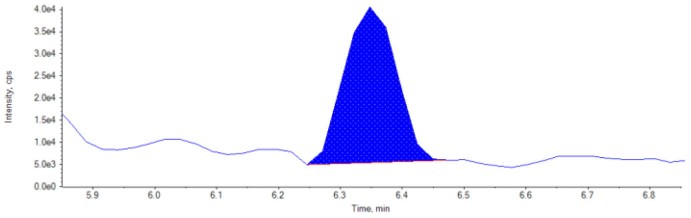

NT-2

RT (Exp. RT):	6.35 (6.35) min
Calculated Conc:	7.56 ng/mL
Area:	214000.
Area Ratio:	3.82e-001
Sample Type:	(Unknown)

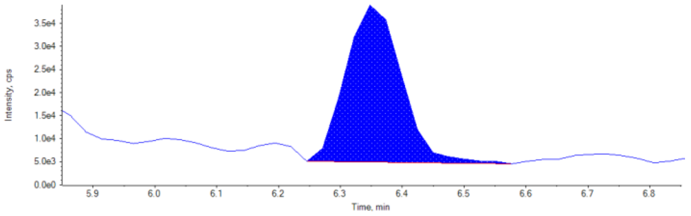

NT-3

RT (Exp. RT):	6.34 (6.35) min
Calculated Conc:	7.13 ng/mL
Area:	201000.
Area Ratio:	3.60e-001
Sample Type:	(Unknown)

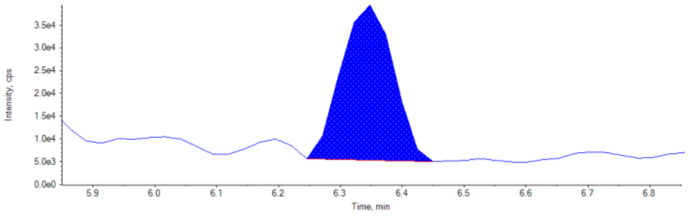

MT-1

RT (Exp. RT):	6.35 (6.35) min
Calculated Conc:	26.6 ng/mL
Area:	713000.
Area Ratio:	1.34e+000
Sample Type:	(Unknown)

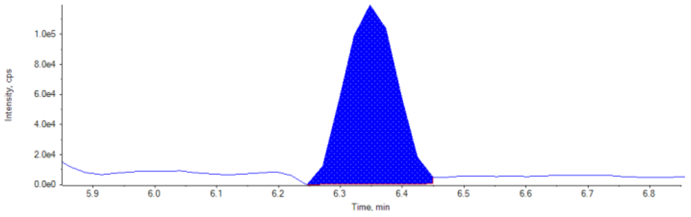

MT-2

RT (Exp. RT):	6.34 (6.35) min
Calculated Conc:	29.9 ng/mL
Area:	782000.
Area Ratio:	1.51e+000
Sample Type:	(Unknown)

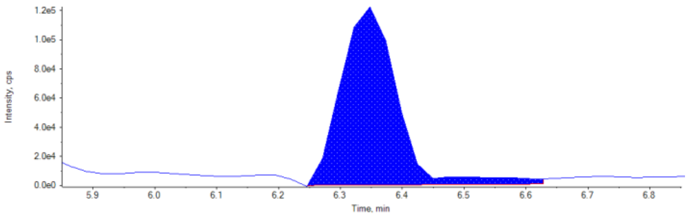

MT-3

RT (Exp. RT):	6.35 (6.35) min
Calculated Conc:	30.7 ng/mL
Area:	766000.
Area Ratio:	1.55e+000
Sample Type:	(Unknown)

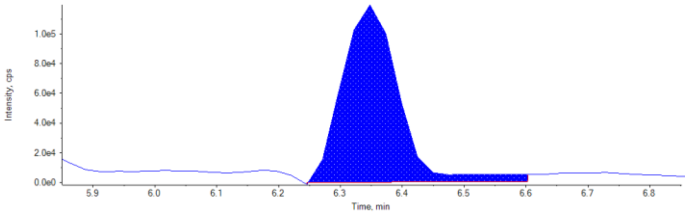

HT-1

RT (Exp. RT):	6.34 (6.35) min
Calculated Conc:	10.9 ng/mL
Area:	277000.
Area Ratio:	5.51e-001
Sample Type:	(Unknown)

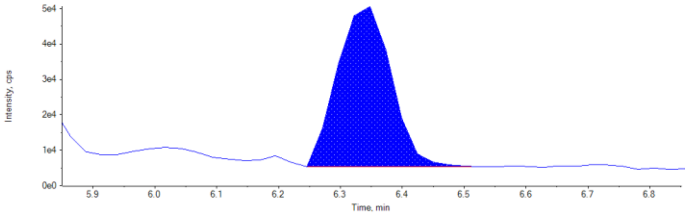

HT-2

RT (Exp. RT):	6.35 (6.35) min
Calculated Conc:	11.2 ng/mL
Area:	271000.
Area Ratio:	5.67e-001
Sample Type:	(Unknown)

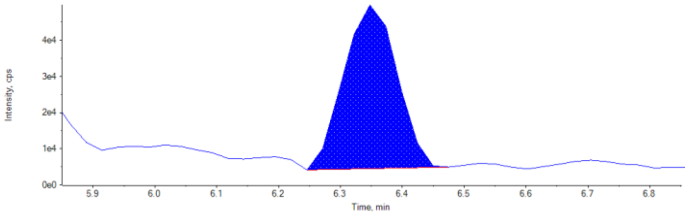

HT-3

RT (Exp. RT): 6.34 (6.35) min

Calculated Conc: 10.8 ng/mL

Area: 277000.

Area Ratio: 5.43e-001

Sample Type: (Unknown)

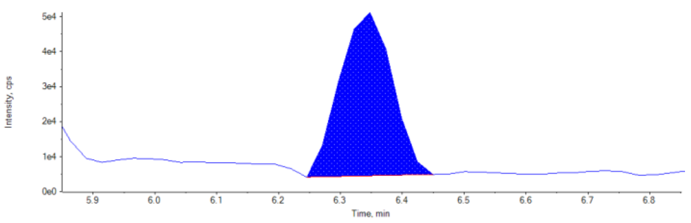

Analyte: TZ 1

NT-1

RT (Exp. RT): 3.61 (3.55) min

Calculated Conc: N/A ng/mL

Area: 85500.

Area Ratio: 2.17e-002

Sample Type: (Unknown)

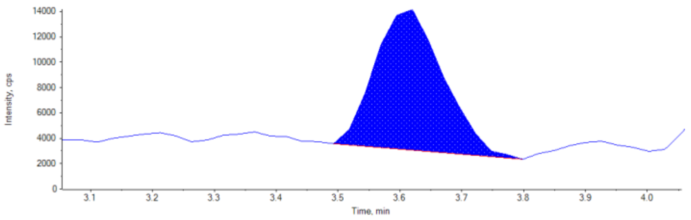

NT-2

RT (Exp. RT): 3.64 (3.55) min

Calculated Conc: N/A ng/mL

Area: 90000.

Area Ratio: 2.43e-002

Sample Type: (Unknown)

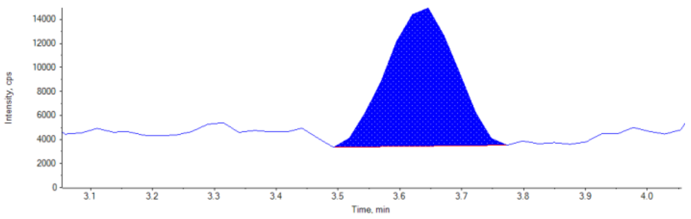

NT-3

RT (Exp. RT): 3.66 (3.55) min

Calculated Conc: N/A ng/mL

Area: 57000.

Area Ratio: 1.60e-002

Sample Type: (Unknown)

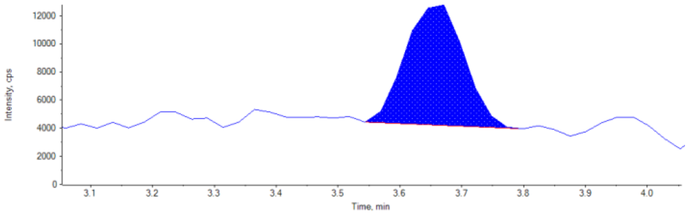

LT-1

RT (Exp. RT): 3.66 (3.55) min

Calculated Conc: 0.428 ng/mL

Area: 157000.

Area Ratio: 5.45e-002

Sample Type: (Unknown)

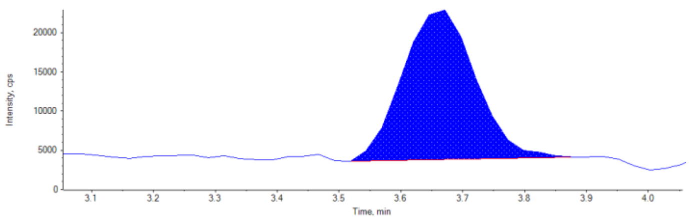

LT-2

RT (Exp. RT): 3.68 (3.55) min

Calculated Conc: 0.511 ng/mL

Area: 165000.

Area Ratio: 6.05e-002

Sample Type: (Unknown)

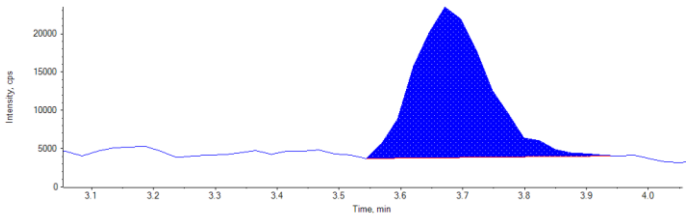

LT-3

RT (Exp. RT): 3.66 (3.55) min

Calculated Conc: 0.522 ng/mL

Area: 177000.

Area Ratio: 6.13e-002

Sample Type: (Unknown)

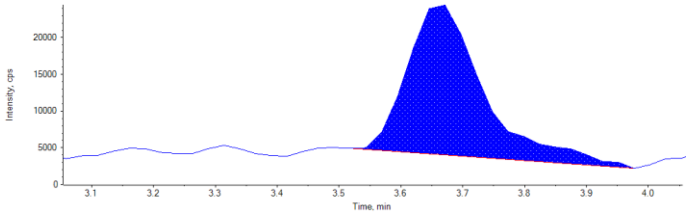

MT-1

RT (Exp. RT): 3.67 (3.55) min

Calculated Conc: 0.406 ng/mL

Area: 144000.

Area Ratio: 5.29e-002

Sample Type: (Unknown)

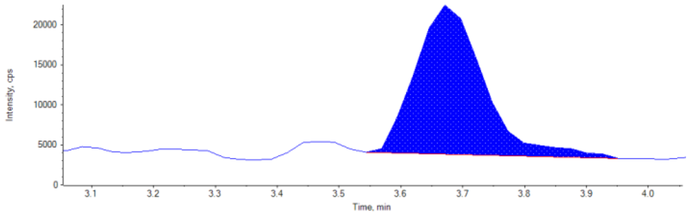

MT-2

RT (Exp. RT): 3.67 (3.55) min

Calculated Conc: 0.408 ng/mL

Area: 163000.

Area Ratio: 5.30e-002

Sample Type: (Unknown)

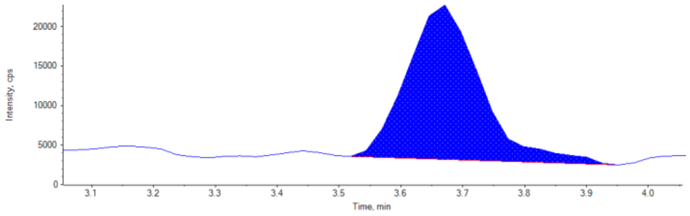

MT-3

RT (Exp. RT): 3.64 (3.55) min

Calculated Conc: 0.368 ng/mL

Area: 138000.

Area Ratio: 5.01e-002

Sample Type: (Unknown)

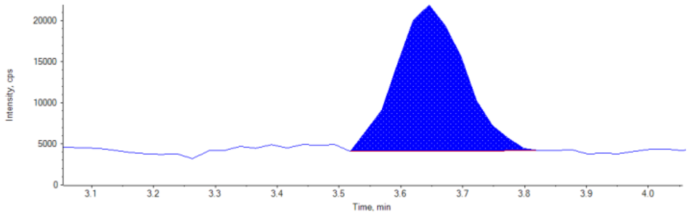

HT-1

RT (Exp. RT): 3.66 (3.55) min

Calculated Conc: 0.546 ng/mL

Area: 165000.

Area Ratio: 6.30e-002

Sample Type: (Unknown)

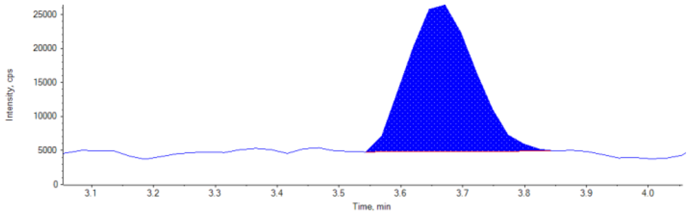

HT-2

RT (Exp. RT): 3.66 (3.55) min

Calculated Conc: 0.603 ng/mL

Area: 173000.

Area Ratio: 6.71e-002

Sample Type: (Unknown)

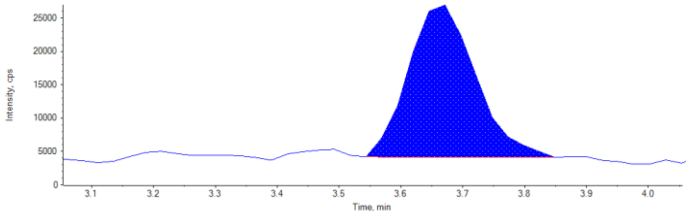

HT-3

RT (Exp. RT): 3.67 (3.55) min

Calculated Conc: 0.493 ng/mL

Area: 154000.

Area Ratio: 5.92e-002

Sample Type: (Unknown)

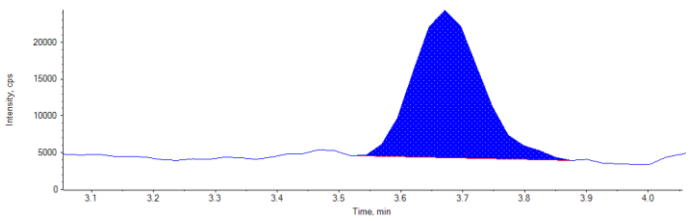

Analyte: MEJA 2

NT-1

RT (Exp. RT): 0.00 (9.29) min

Calculated Conc: N/A ng/mL

Area: 0.00

Area Ratio: 0.00e+000

Sample Type: (Unknown)

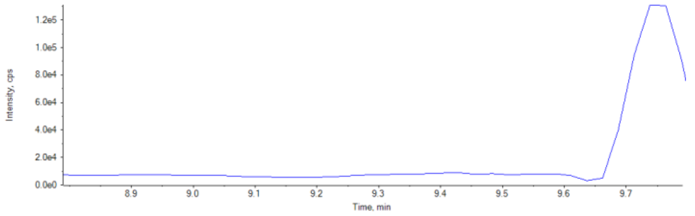

NT-1

RT (Exp. RT): 9.39 (9.29) min

Calculated Conc: N/A ng/mL

Area: 55300.

Area Ratio: 5.45e-002

Sample Type: (Unknown)

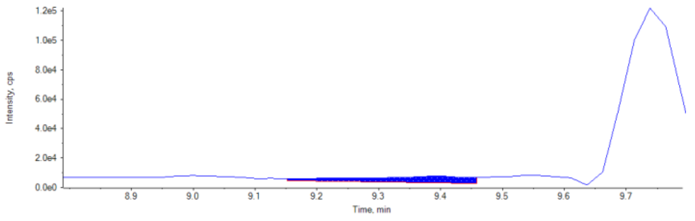

NT-3

RT (Exp. RT): 0.00 (9.29) min

Calculated Conc: N/A ng/mL

Area: 0.00

Area Ratio: 0.00e+000

Sample Type: (Unknown)

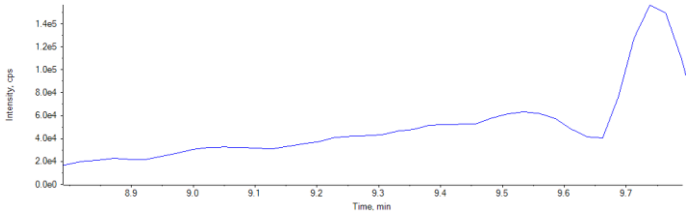

LT-1

RT (Exp. RT):	0.00 (9.29) min
Calculated Conc:	N/A ng/mL
Area:	0.00
Area Ratio:	0.00e+000
Sample Type:	(Unknown)

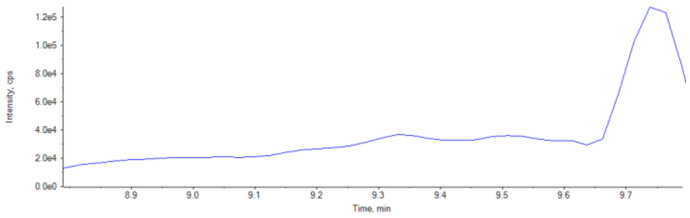

LT-2

RT (Exp. RT):	9.34 (9.29) min
Calculated Conc:	N/A ng/mL
Area:	237000.
Area Ratio:	4.23e-001
Sample Type:	(Unknown)

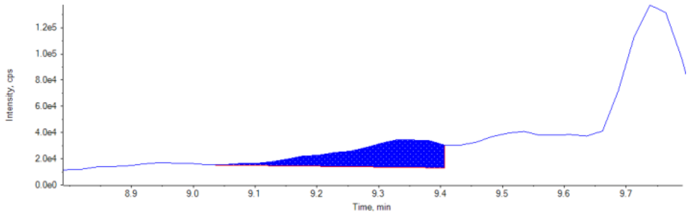

LT-3

RT (Exp. RT):	9.27 (9.29) min
Calculated Conc:	N/A ng/mL
Area:	4930.
Area Ratio:	9.02e-003
Sample Type:	(Unknown)

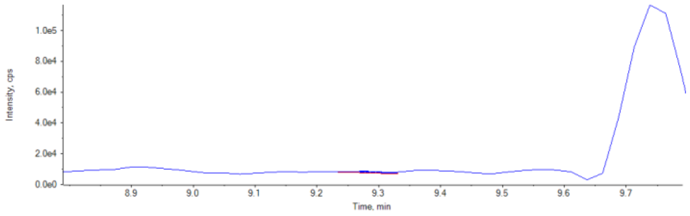

MT-1

RT (Exp. RT):	0.00 (9.29) min
Calculated Conc:	N/A ng/mL
Area:	0.00
Area Ratio:	0.00e+000
Sample Type:	(Unknown)

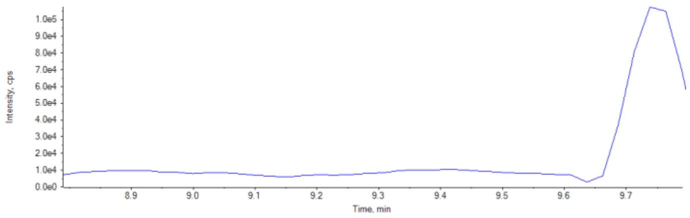

MT-2

RT (Exp. RT): 0.00 (9.29) min

Calculated Conc: N/A ng/mL

Area: 0.00

Area Ratio: 0.00e+000

Sample Type: (Unknown)

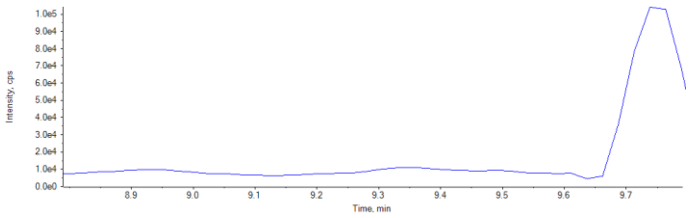

MT-3

RT (Exp. RT): 9.29 (9.29) min

Calculated Conc: N/A ng/mL

Area: 105000.

Area Ratio: 2.14e-001

Sample Type: (Unknown)

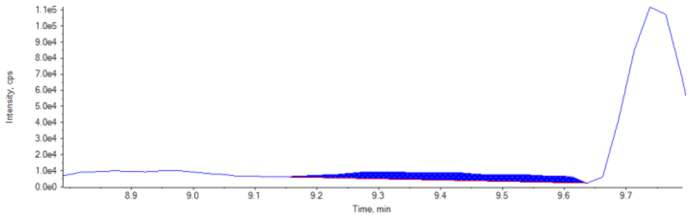

HT-1

RT (Exp. RT): 9.37 (9.29) min

Calculated Conc: N/A ng/mL

Area: 59800.

Area Ratio: 1.18e-001

Sample Type: (Unknown)

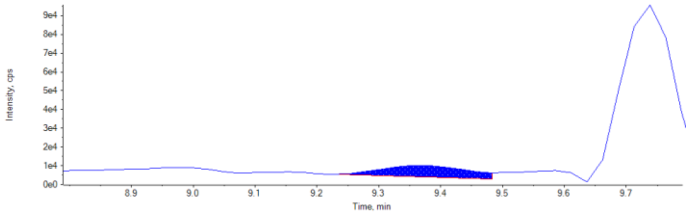

HT-2

RT (Exp. RT): 0.00 (9.29) min

Calculated Conc: N/A ng/mL

Area: 0.00

Area Ratio: 0.00e+000

Sample Type: (Unknown)

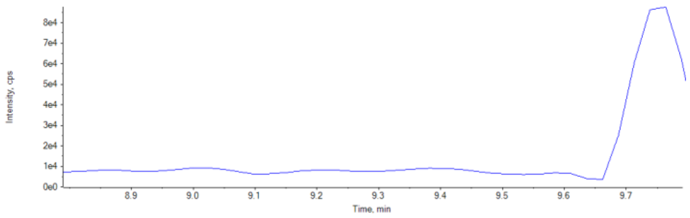

HT-3

RT (Exp. RT): 0.00 (9.29) min

Calculated Conc: N/A ng/mL

Area: 0.00

Area Ratio: 0.00e+000

Sample Type: (Unknown)

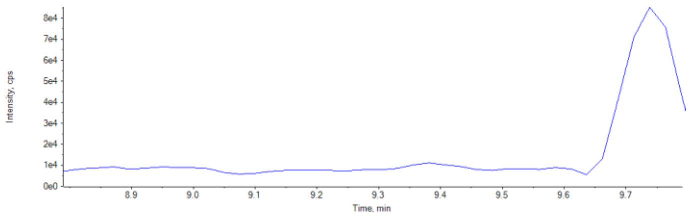

Analyte: ABA 1

NT-1

RT (Exp. RT): 7.30 (7.31) min

Calculated Conc: 4.20 ng/mL

Area: 75300.

Area Ratio: 1.82e+000

Sample Type: (Unknown)

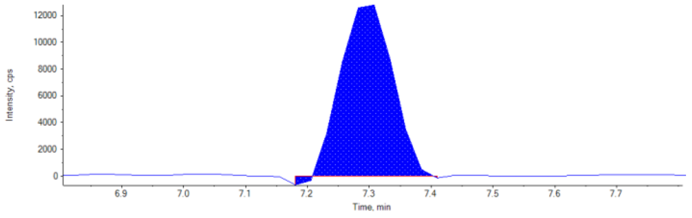

NT-2

RT (Exp. RT): 7.29 (7.31) min

Calculated Conc: 3.41 ng/mL

Area: 53900.

Area Ratio: 1.48e+000

Sample Type: (Unknown)

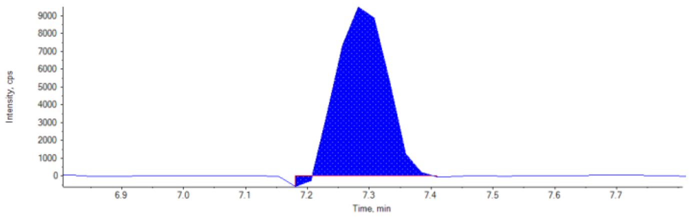

NT-3

RT (Exp. RT): 7.30 (7.31) min

Calculated Conc: 3.86 ng/mL

Area: 50600.

Area Ratio: 1.67e+000

Sample Type: (Unknown)

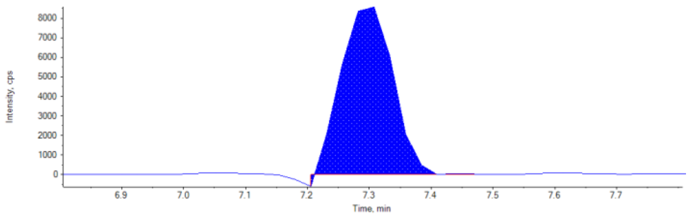

LT-1

RT (Exp. RT):	7.29 (7.31) min
Calculated Conc:	8.68 ng/mL
Area:	28700.
Area Ratio:	3.72e+000
Sample Type:	(Unknown)

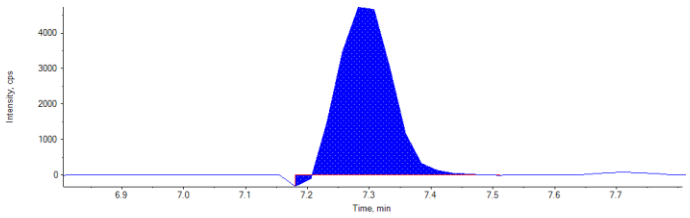

LT-2

RT (Exp. RT):	7.30 (7.31) min
Calculated Conc:	5.36 ng/mL
Area:	24000.
Area Ratio:	2.31e+000
Sample Type:	(Unknown)

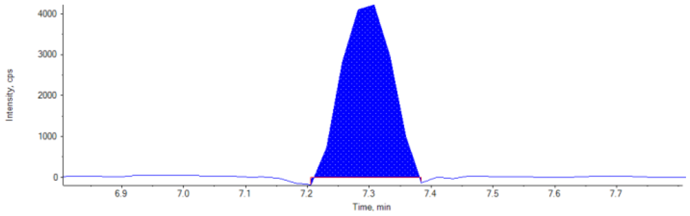

LT-3

RT (Exp. RT):	7.29 (7.31) min
Calculated Conc:	6.57 ng/mL
Area:	23300.
Area Ratio:	2.83e+000
Sample Type:	(Unknown)

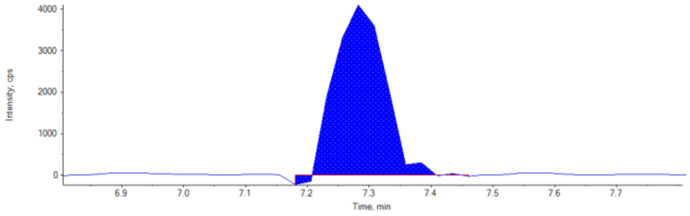

MT-1

RT (Exp. RT):	7.30 (7.31) min
Calculated Conc:	4.41 ng/mL
Area:	15500.
Area Ratio:	1.91e+000
Sample Type:	(Unknown)

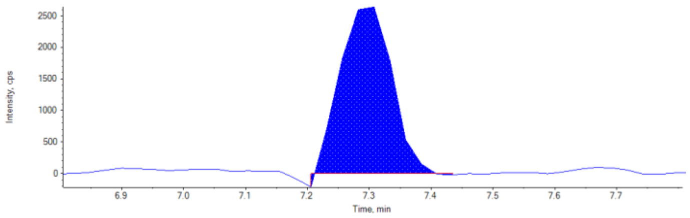

MT-2

RT (Exp. RT):	7.30 (7.31) min
Calculated Conc:	4.63 ng/mL
Area:	13800.
Area Ratio:	2.00e+000
Sample Type:	(Unknown)

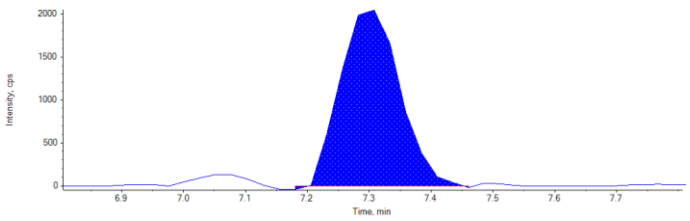

MT-3

RT (Exp. RT):	7.30 (7.31) min
Calculated Conc:	3.97 ng/mL
Area:	12900.
Area Ratio:	1.72e+000
Sample Type:	(Unknown)

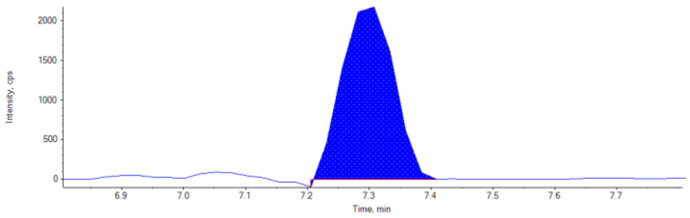

HT-1

RT (Exp. RT):	7.28 (7.31) min
Calculated Conc:	6.08 ng/mL
Area:	22000.
Area Ratio:	2.62e+000
Sample Type:	(Unknown)

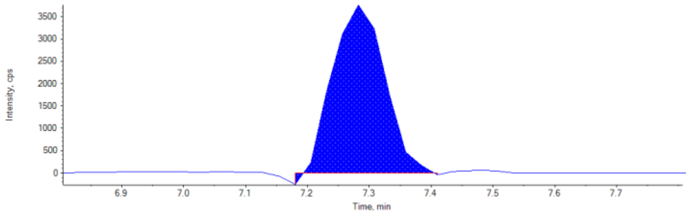

HT-2

RT (Exp. RT):	7.30 (7.31) min
Calculated Conc:	6.19 ng/mL
Area:	20500.
Area Ratio:	2.67e+000
Sample Type:	(Unknown)

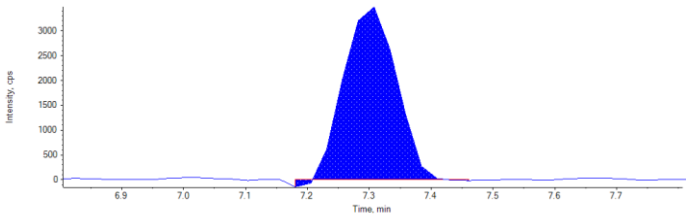

HT-3

RT (Exp. RT):	7.29 (7.31) min
Calculated Conc:	8.06 ng/mL
Area:	22200.
Area Ratio:	3.46e+000
Sample Type:	(Unknown)

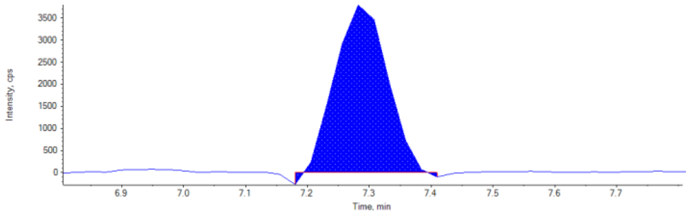

Analyte: GA3-1

NT-1

RT (Exp. RT):	5.76 (5.87) min
Calculated Conc:	2.16 ng/mL
Area:	260000.
Area Ratio:	1.97e+000
Sample Type:	(Unknown)

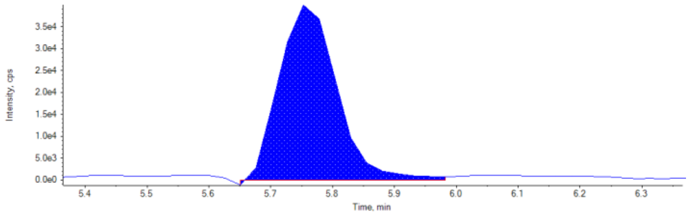

NT-2

RT (Exp. RT):	5.76 (5.87) min
Calculated Conc:	2.42 ng/mL
Area:	249000.
Area Ratio:	2.20e+000
Sample Type:	(Unknown)

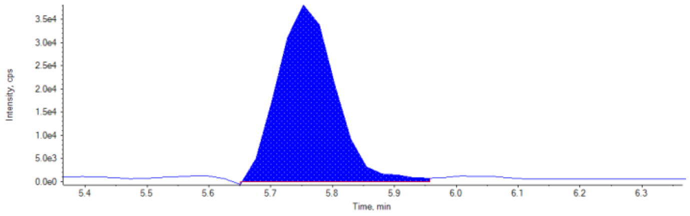

NT-3

RT (Exp. RT):	5.76 (5.87) min
Calculated Conc:	2.24 ng/mL
Area:	243000.
Area Ratio:	2.04e+000
Sample Type:	(Unknown)

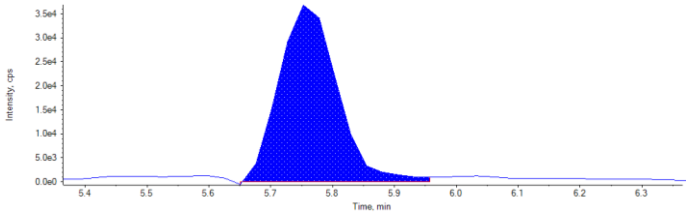

LT-1

RT (Exp. RT):	5.78 (5.87) min
Calculated Conc:	15.8 ng/mL
Area:	1070000.
Area Ratio:	1.43e+001
Sample Type:	(Unknown)

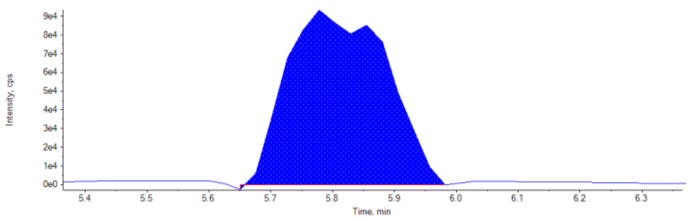

LT-2

RT (Exp. RT):	5.76 (5.87) min
Calculated Conc:	17.1 ng/mL
Area:	1000000.
Area Ratio:	1.55e+001
Sample Type:	(Unknown)

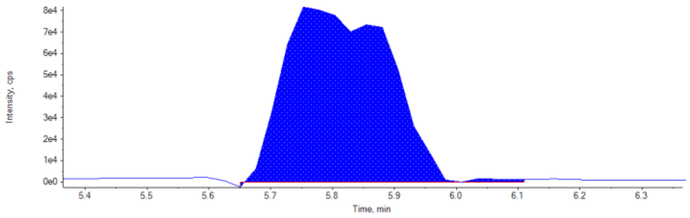

LT-3

RT (Exp. RT):	5.84 (5.87) min
Calculated Conc:	16.0 ng/mL
Area:	950000.
Area Ratio:	1.45e+001
Sample Type:	(Unknown)

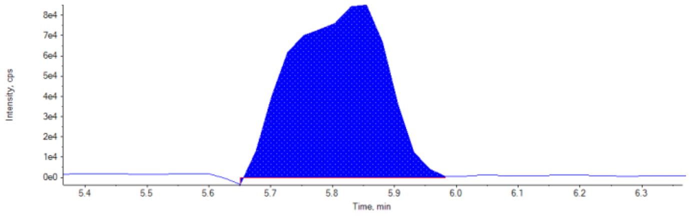

MT-1

RT (Exp. RT):	5.76 (5.87) min
Calculated Conc:	10.1 ng/mL
Area:	644000.
Area Ratio:	9.11e+000
Sample Type:	(Unknown)

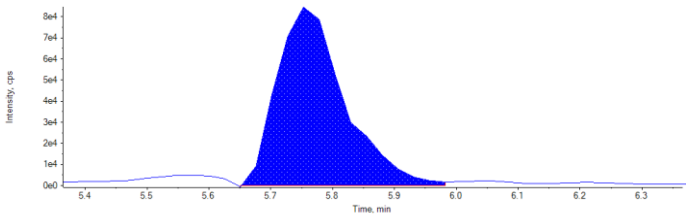

MT-2

RT (Exp. RT): 5.76 (5.87) min
Calculated Conc: 10.5 ng/mL
Area: 671000.
Area Ratio: 9.48e+000
Sample Type: (Unknown)

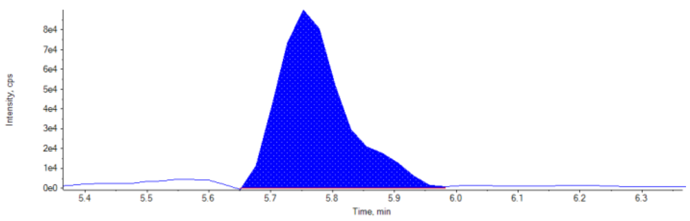

MT-3

RT (Exp. RT): 5.76 (5.87) min
Calculated Conc: 10.2 ng/mL
Area: 565000.
Area Ratio: 9.27e+000
Sample Type: (Unknown)

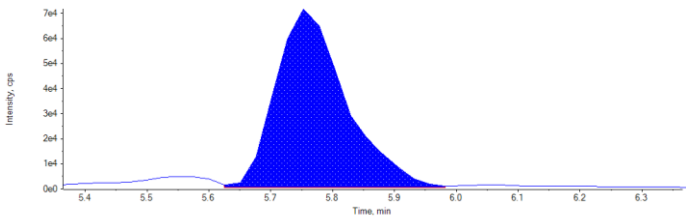

HT-1

RT (Exp. RT): 5.85 (5.87) min
Calculated Conc: 32.7 ng/mL
Area: 1830000.
Area Ratio: 2.96e+001
Sample Type: (Unknown)

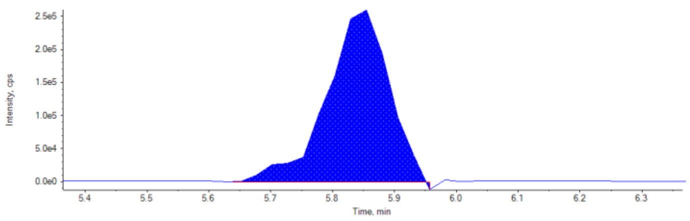

HT-2

RT (Exp. RT): 5.87 (5.87) min
Calculated Conc: 32.3 ng/mL
Area: 1910000.
Area Ratio: 2.93e+001
Sample Type: (Unknown)

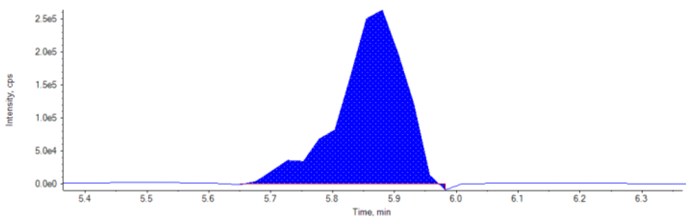

HT-3	
RT (Exp. RT):	5.86 (5.87) min
Calculated Conc:	42.1 ng/mL
Area:	2020000.
Area Ratio:	3.81e+001
Sample Type:	(Unknown)

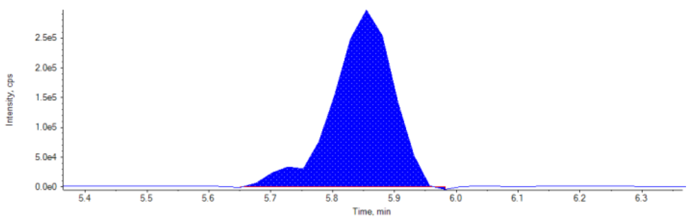

2. Standard raw data

Analyte: IAA 1

S0.2	
RT (Exp. RT):	6.33 (6.35) min
Calculated Conc:	0.195 ng/mL
Area:	24600.
Area Ratio:	1.08e-002
Sample Type:	(Standard)

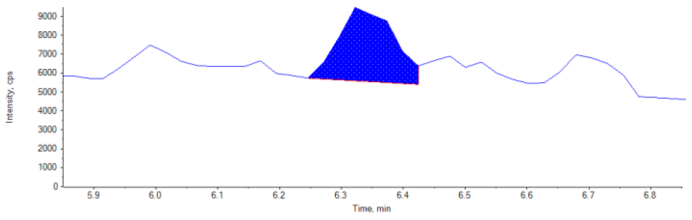

S0.5	
RT (Exp. RT):	6.34 (6.35) min
Calculated Conc:	0.512 ng/mL
Area:	57800.
Area Ratio:	2.67e-002
Sample Type:	(Standard)

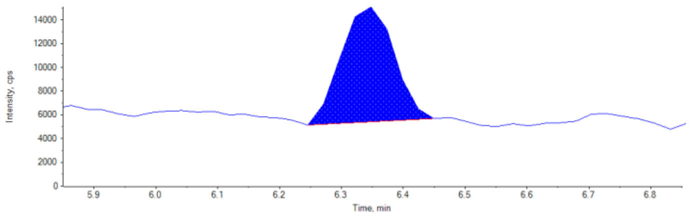

S2

RT (Exp. RT): 6.35 (6.35) min

Calculated Conc: 2.23 ng/mL

Area: 226000.

Area Ratio: 1.14e-001

Sample Type: (Standard)

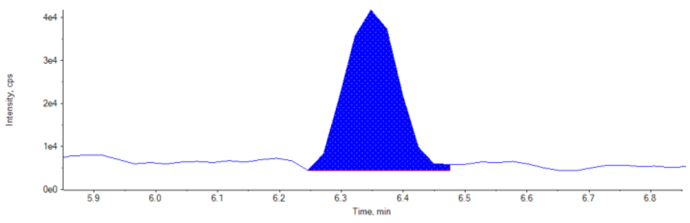

S5

RT (Exp. RT): 6.35 (6.35) min

Calculated Conc: 5.52 ng/mL

Area: 546000.

Area Ratio: 2.79e-001

Sample Type: (Standard)

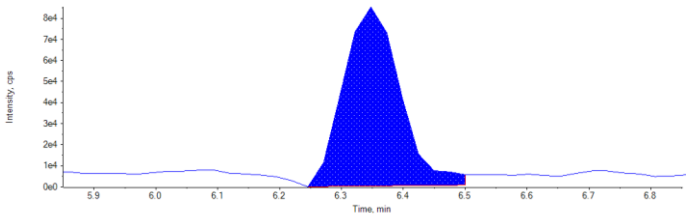

S20

RT (Exp. RT): 6.34 (6.35) min

Calculated Conc: 18.8 ng/mL

Area: 1930000.

Area Ratio: 9.47e-001

Sample Type: (Standard)

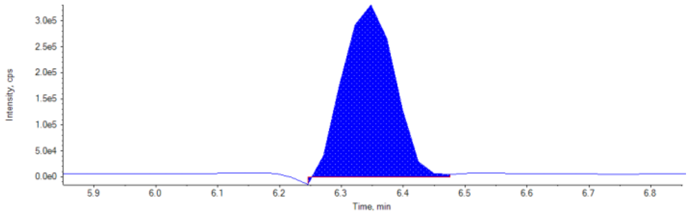

S50

RT (Exp. RT): 6.35 (6.35) min

Calculated Conc: 47.0 ng/mL

Area: 4600000.

Area Ratio: 2.37e+000

Sample Type: (Standard)

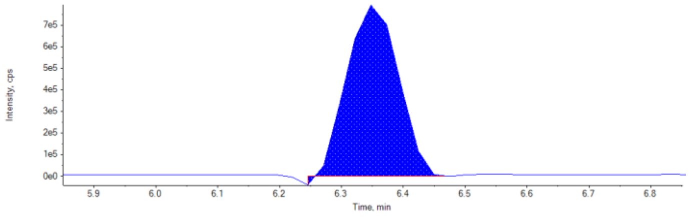

S200	
RT (Exp. RT):	6.35 (6.35) min
Calculated Conc:	180. ng/mL
Area:	19100000.
Area Ratio:	9.07e+000
Sample Type:	(Standard)

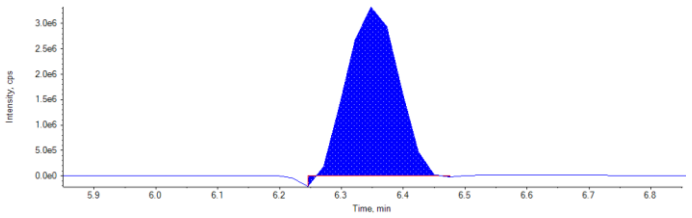

nalyte: TZ 1

S0.2	
RT (Exp. RT):	3.57 (3.55) min
Calculated Conc:	0.200 ng/mL
Area:	373000.
Area Ratio:	3.80e-002
Sample Type:	(Standard)

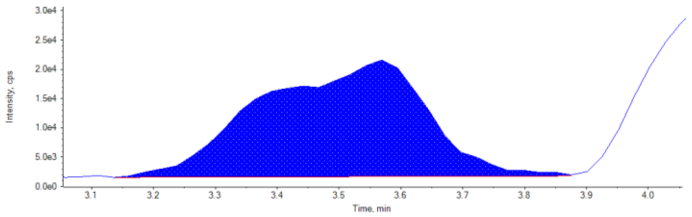

S5	
RT (Exp. RT):	3.56 (3.55) min
Calculated Conc:	4.77 ng/mL
Area:	3210000.
Area Ratio:	3.68e-001
Sample Type:	(Standard)

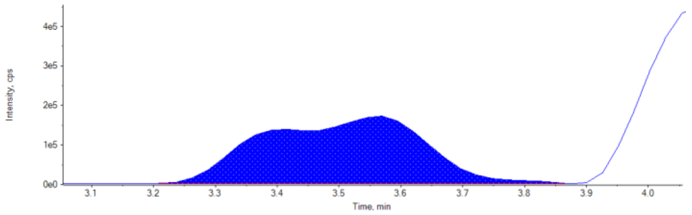

S20	
RT (Exp. RT):	3.58 (3.55) min
Calculated Conc:	20.1 ng/mL
Area:	12100000.
Area Ratio:	1.47e+000
Sample Type:	(Standard)

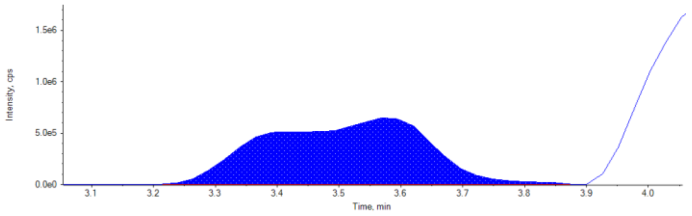

S50	
RT (Exp. RT):	3.56 (3.55) min
Calculated Conc:	52.0 ng/mL
Area:	29800000.
Area Ratio:	3.77e+000
Sample Type:	(Standard)

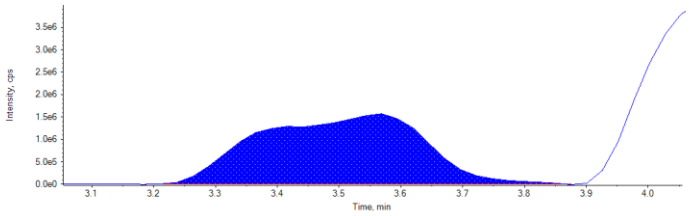

Analyte: MEJA 2

S0.2	
RT (Exp. RT):	9.27 (9.29) min
Calculated Conc:	0.200 ng/mL
Area:	28300.
Area Ratio:	1.86e-002
Sample Type:	(Standard)

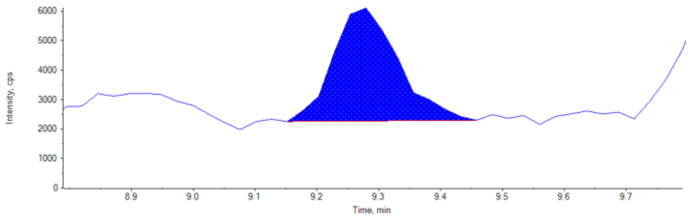

S0.5	
RT (Exp. RT):	9.28 (9.29) min
Calculated Conc:	0.502 ng/mL
Area:	46100.
Area Ratio:	3.17e-002
Sample Type:	(Standard)

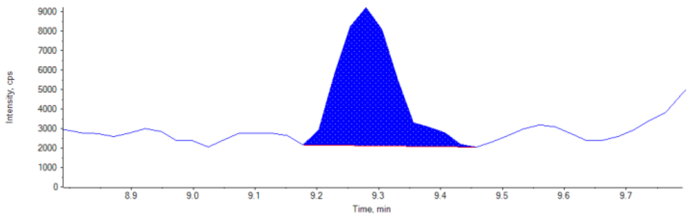

S5

RT (Exp. RT): 9.29 (9.29) min

Calculated Conc: 5.12 ng/mL

Area: 369000.

Area Ratio: 2.32e-001

Sample Type: (Standard)

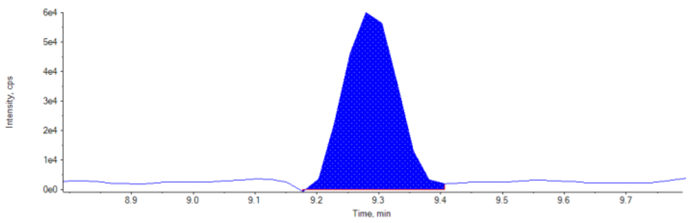

S20

RT (Exp. RT): 9.28 (9.29) min

Calculated Conc: 20.9 ng/mL

Area: 1310000.

Area Ratio: 9.14e-001

Sample Type: (Standard)

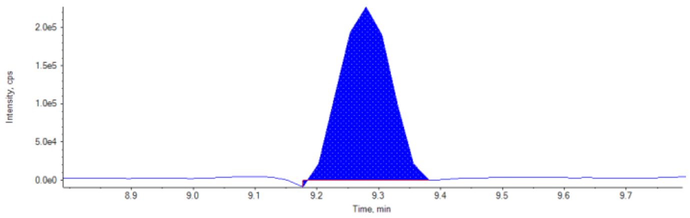

S50

RT (Exp. RT): 9.28 (9.29) min

Calculated Conc: 46.6 ng/mL

Area: 3450000.

Area Ratio: 2.03e+000

Sample Type: (Standard)

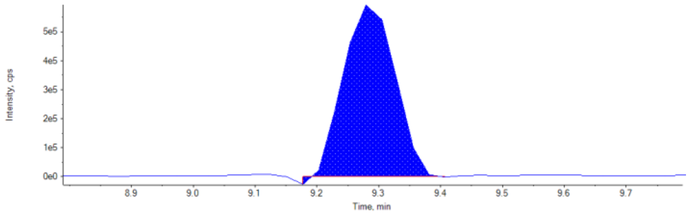

Analyte: ABA 1

S0.1

RT (Exp. RT):	7.30 (7.31) min
Calculated Conc:	0.102 ng/mL
Area:	8490.
Area Ratio:	7.12e-002
Sample Type:	(Standard)

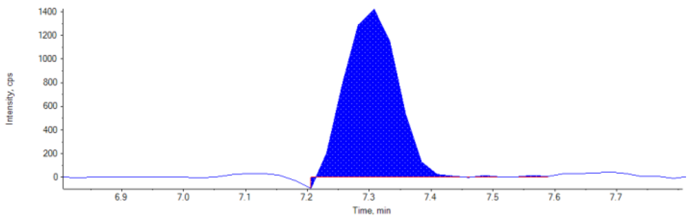

S0.2

RT (Exp. RT):	7.30 (7.31) min
Calculated Conc:	0.195 ng/mL
Area:	14900.
Area Ratio:	1.11e-001
Sample Type:	(Standard)

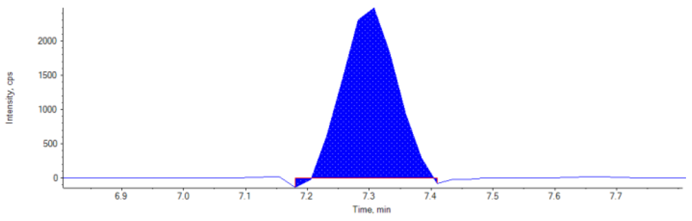

S0.5

RT (Exp. RT): 7.30 (7.31) min

Calculated Conc: 0.472 ng/mL

Area: 28600.

Area Ratio: 2.29e-001

Sample Type: (Standard)

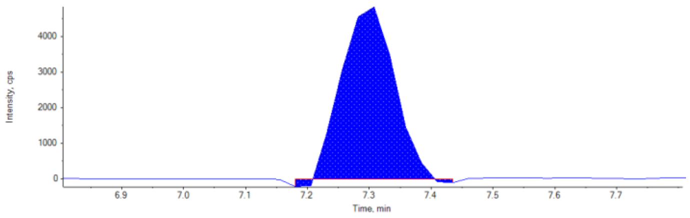

S2

RT (Exp. RT): 7.30 (7.31) min

Calculated Conc: 1.99 ng/mL

Area: 96200.

Area Ratio: 8.75e-001

Sample Type: (Standard)

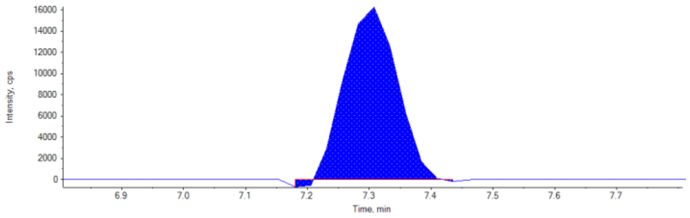

S5

RT (Exp. RT): 7.30 (7.31) min

Calculated Conc: 4.88 ng/mL

Area: 237000.

Area Ratio: 2.11e+000

Sample Type: (Standard)

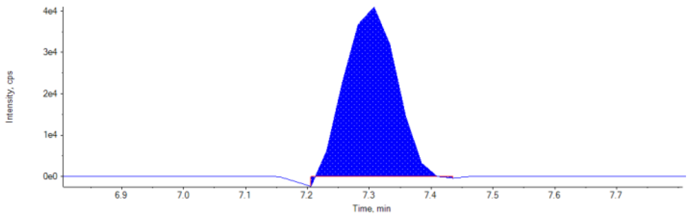

S20

RT (Exp. RT): 7.30 (7.31) min

Calculated Conc: 20.1 ng/mL

Area: 864000.

Area Ratio: 8.60e+000

Sample Type: (Standard)

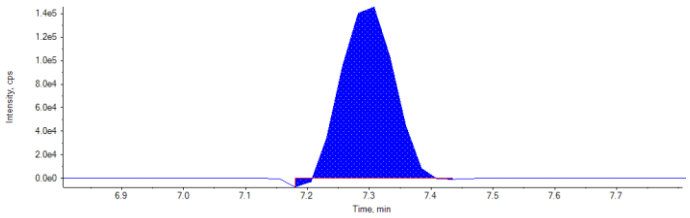

S50

RT (Exp. RT): 7.30 (7.31) min

Calculated Conc: 53.9 ng/mL

Area: 2270000.

Area Ratio: 2.30e+001

Sample Type: (Standard)

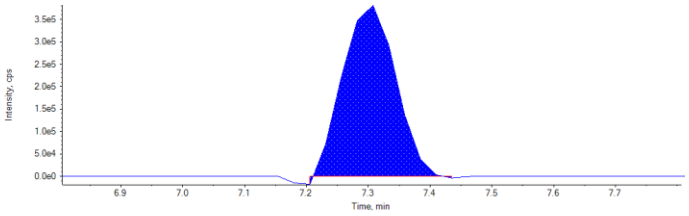

S200

RT (Exp. RT): 7.31 (7.31) min

Calculated Conc: 200. ng/mL

Area: 8870000.

Area Ratio: 8.54e+001

Sample Type: (Standard)

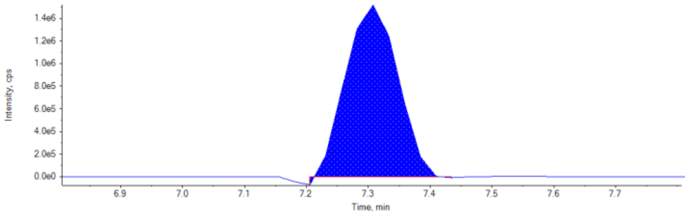

Analyte: GA3-1

S0.1

RT (Exp. RT): 5.87 (5.87) min

Calculated Conc: 0.0990 ng/mL

Area: 23000.

Area Ratio: 1.04e-001

Sample Type: (Standard)

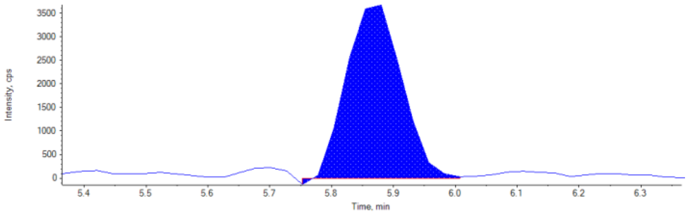

S0.2

RT (Exp. RT): 5.86 (5.87) min

Calculated Conc: 0.190 ng/mL

Area: 49200.

Area Ratio: 1.86e-001

Sample Type: (Standard)

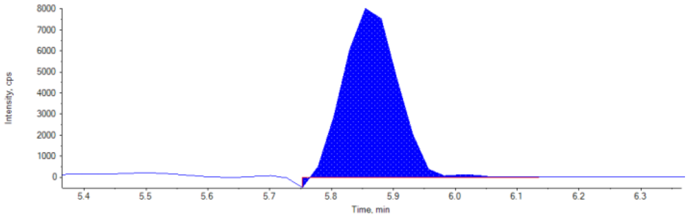

S0.5

RT (Exp. RT):	5.86 (5.87) min
Calculated Conc:	0.581 ng/mL
Area:	114000.
Area Ratio:	5.40e-001
Sample Type:	(Standard)

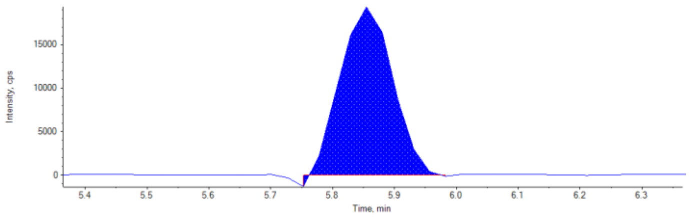

S2

RT (Exp. RT):	5.86 (5.87) min
Calculated Conc:	2.08 ng/mL
Area:	425000.
Area Ratio:	1.90e+000
Sample Type:	(Standard)

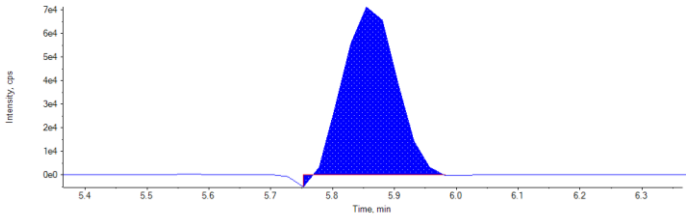

S5

RT (Exp. RT):	5.86 (5.87) min
Calculated Conc:	4.91 ng/mL
Area:	980000.
Area Ratio:	4.46e+000
Sample Type:	(Standard)

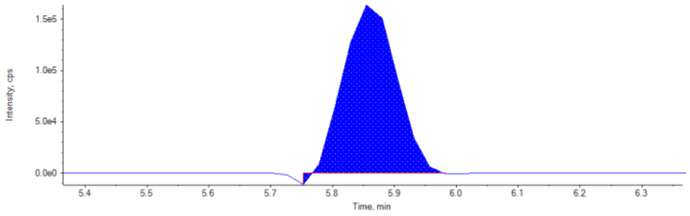

S20

RT (Exp. RT):	5.86 (5.87) min
Calculated Conc:	19.8 ng/mL
Area:	3870000.
Area Ratio:	1.80e+001
Sample Type:	(Standard)

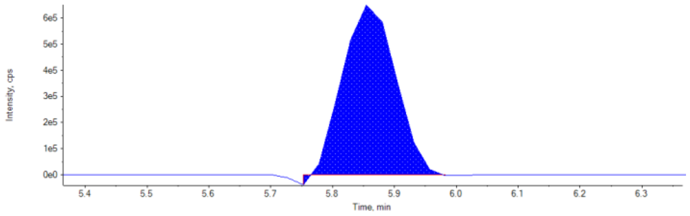

S50<br><br>RT (Exp. RT): 5.86 (5.87) min<br>Calculated Conc: 44.1 ng/mL<br>Area: 9360000.<br>Area Ratio: 3.99e+001<br>Sample Type: (Standard)	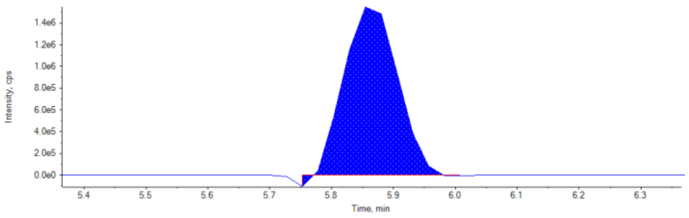
